# Chemical composition and biological activities of essential oils of seven Cultivated *Apiaceae* species

**DOI:** 10.1038/s41598-024-60810-3

**Published:** 2024-05-02

**Authors:** Sercan Önder, Çağdaş Deniz Periz, Seyhan Ulusoy, Sabri Erbaş, Damla Önder, Muhammet Tonguç

**Affiliations:** 1 Department of Agricultural Biotechnology, Faculty of Agriculture, Isparta University of Applied Sciences, 32200 Isparta, Türkiye; 2 Department of Biology, Faculty of Engineering and Natural Sciences, Süleyman Demirel University, 32260 Isparta, Türkiye; 3 Department of Field Crops, Faculty of Agriculture, Isparta University of Applied Sciences, 32200 Isparta, Türkiye

**Keywords:** Plant biotechnology, Plant biotechnology

## Abstract

The *Apiaceae* family contains many species used as food, spice and medicinal purposes. Different parts of plants including seeds could be used to obtain essential (EO) oils from members of the *Apiaceae* family. In the present study, EOs were components obtained through hydrodistillation from the seeds of anise (*Pimpinella anisum*), carrot (*Daucus carota*), celery (*Apium graveolens*), dill (*Anethum graveolens*), coriander (*Coriandrum sativum*), fennel (*Foeniculum vulgare*), and cumin (*Cuminum cyminum*). EO constituents were determined with Gas Chromatography/Mass Spectrometry (GC–MS) and Gas Chromatography/Flame Ionization Detector (GC-FID) and their antioxidant capacities were determined with the cupric reducing antioxidant capacity (CUPRAC) and 2,2-diphenyl-1-picryl-hydrazyl-hydrate (DPPH) methods. The antimicrobial activity of EOs were tested against four pathogenic bacteria. Phenylpropanoids in anise (94.87%) and fennel (92.52%), oxygenated monoterpenes in dill (67.59%) and coriander (98.96%), monoterpene hydrocarbons in celery (75.42%), mono- (45.42%) and sesquiterpene- (43.25%) hydrocarbons in carrots, monoterpene hydrocarbon (34.30%) and aromatic hydrocarbons (32.92%) in cumin were the major compounds in the EOs. Anethole in anise and fennel, carotol in carrot, limonene in celery, carvone in dill, linalool in coriander, and cumin aldehyde in cumin were predominant compounds in these EOs. The high hydrocarbon content in cumin EO gave high CUPRAC activity (89.07 µmol Trolox g^−1^), and the moderate monoterpene hydrocarbon and oxygenated monoterpene content in dill EO resulted in higher DPPH activity (9.86 µmol Trolox g^−1^). The in vitro antibacterial activity of EOs against *Bacillus cereus*, *Staphylococcus aureus*, *Pseudomonas aeruginosa* and *Escherichia coli* was evaluated using the agar diffusion method and the minimum bactericidal concentration was determined. Coriander, cumin and dill EOs showed inhibitory effect against all tested strains except *P. aeruginosa*. While fennel and celery EOs were effective against *E. coli* and *B. cereus* strains, respectively, anise and carrot EOs did not show any antibacterial effect against the tested bacteria. Hierarchical Cluster Analysis (HCA) produced four groups based on EO constituents of seven species. The potential adoption of the cultivated *Apiaceae* species for EO extraction could be beneficial for the wild species that are endangered by over collection and consumption.

## Introduction

The *Apiaceae* or *Umbelliferae* is a large plant family containing 434 genera and 3780 hollow-stemmed aromatic plant species^1^. The common traits of the *Apiaceae* family are hollow stems, inflorescences with simple or compound umbels, small flowers, indehiscent fruits and seeds with oil channels^1,2^. Anise (*Pimpinella anisum* L.), carrot (*Daucus carota* L.), celery (*Apium graveolens* L.), dill (*Anethum graveolens* L.), coriander (*Coriandrum sativum* L.), fennel (*Foeniculum vulgare* Mill.) and cumin (*Cuminum cyminum* L.) are among the cultivated members of the *Apiaceae* family that are widely used for food, cosmetic and therapeutic purposes. Anise, carrot, celery, dill, coriander, fennel and cumin essential oils (EOs) are also used in traditional medicine for the treatment of various illnesses due to their beneficial effects on rheumatism, back pain, neurological disorders, appetite, nutrient absorption, microbiota and oxidative state^3,4^. Use of cultivated *Apiaceae* species to obtain EOs could also be beneficial for conservation of the wild *Apiaceae* species since they are endangered by over collection from their natural habitats^5,6^.

Plants produced EOs are highly concentrated hydrophobic liquids with distinct aromatic and EO components, which easily evaporate at room temperature. EOs consist of complexes and volatile compounds synthesized as secondary metabolites by medicinal and aromatic plants^3^. EOs protect plants from bacterial, fungal and viral diseases, and prevent oxidative damage of various cellular structures caused by ultraviolet radiation^7^. The distinctive aromatic scent of many plant species is a result of the quantity and composition of EO components they contain. These EOs can be isolated by cold pressing, steam or hydro-distillation from the seeds, leaves, flowers, bark, and roots. However, mericarp glands of *Apiaceae* species contain the highest amount of EOs^1^. Plant EO components are basically grouped into two different classes: terpenoids (monoterpene hydrocarbons, sesquiterpene hydrocarbons, aromatic hydrocarbons and their oxygenated derivatives) and phenylpropanoids (phenols and phenol ethers)^8^. These two chemical classes include phenolic compounds that are hypothesized to be responsible for the antioxidant capacities of EOs^8,9^. Many species of *Apiaceae* family are good sources of EOs, and more than 760 EO compounds belonging to different chemical classes have been identified in *Apiaceae* family^1^.

Emergence of bacterial diseases with multi-resistant strains along with the increased economic burden has created a significant public health problem. Therefore, natural substances like EOs, which have specific and general antibacterial, antimicrobial and antifungal activities, are highly sought-after for the development of effective and novel antibacterial chemicals^10,11^. Moreover, importance of EOs with broad-spectrum antibacterial and antifungal activities has increased, and they are used in food packaging and products as coatings to protect foods and extend their shelf lives because of their antioxidant activities^9,12^. Therefore, the identification of EOs with antimicrobial activity remains an active area of research.

Occurrences of great variation in the production of essential oils have been reported. It was shown that the production of various secondary compounds are influenced by the environmental conditions^13^, locations^14^, elevation^15^, plant parts used^16^, genotypes^17,18^. Therefore, it is common to have different chemotypes within the same species^14,19,20^ and it is necessary to characterize these types for their active molecules and their biological activities^17,19–21^.

The aim of the present study was to obtain and evaluate EOs from different commercially important and widely cultivated *Apiaceae* species as alternative sources of seed EOs. Anise, carrot, celery, dill, coriander, fennel and cumin were used to obtain seed EOs of these species, and EO compounds were characterized. In addition, antioxidant and antibacterial activities of these EOs against common food-borne and human pathogenic bacteria, namely *Bacillus cereus*, *Staphylococcus aureus*, *Pseudomonas aeruginosa* and *Escherichia coli* were also investigated.

## Results

### Chemical composition of EOs

The EO yield was 1.45% for anise, 0.93% for carrot, 1.63% for celery, 3.23% for dill, 0.40% for coriander, 1.95% for fennel and 0.95% for cumin. The GC–MS was used to determine EO components obtained from the seeds of seven species, and the results were summarized in Table 1. The EOs obtained by hydrodistillation had distinct scents and light brown (fennel), brown (anise), light yellow (dill, coriander, celery) and yellow (carrot, cumin) colors. Forty-nine volatile compounds representing 99.97, 99.04, 99.91, 99.01, 100.0, 100.0 and 99.90% of the EOs of anise, carrot, celery, dill, coriander, fennel and cumin were identified, and grouped into eight classes according to their compositions (Aliphatic aldehyde-AlA, monoterpene hydrocarbon-MH, aromatic alcohol-ArAl, aromatic hydrocarbon-ArHy, oxygenated monoterpene-OM, sesquiterpene hydrocarbon-SH, hydrocarbon-H, phenylpropanoid-P).

**Table 1:**
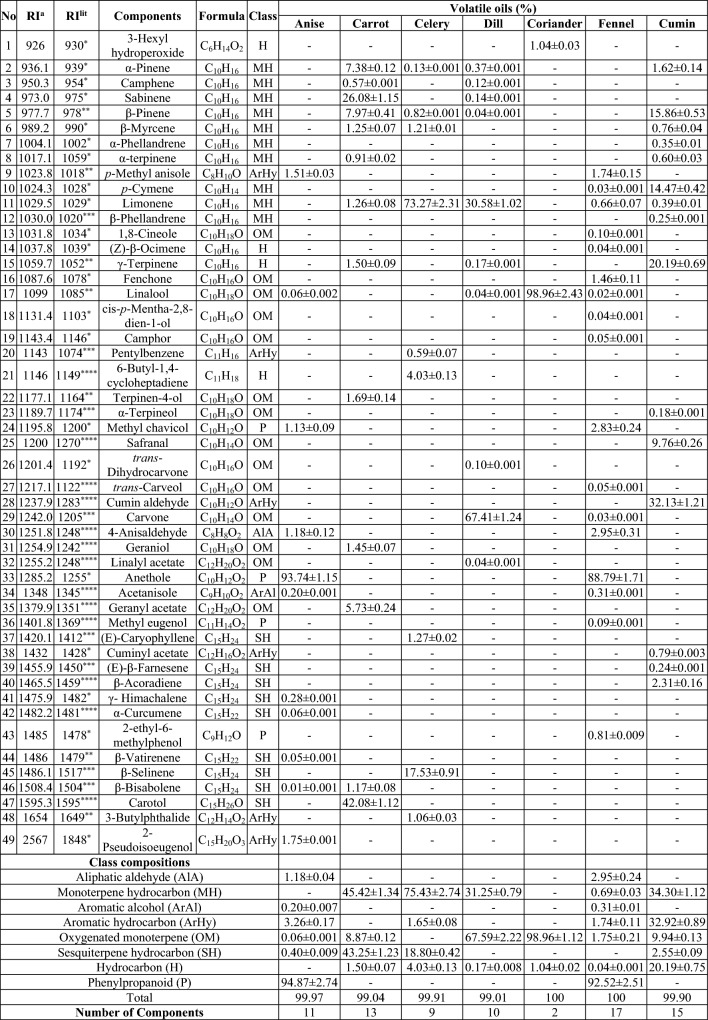
Chemical components of essential oil in seeds of the seven Apiaceae isolated by hydrodistillation.

^a^Retention index were determined on a Restek Rxi®-5Sil MS column using a set of standards consisting of C_6_-C_30_ *n*-alkanes; -:Not detected. *^19^,**^22^,***^23^,****^24^.

In anise and fennel EOs, phenylpropanoids were the most abundant class of volatile compounds representing over 94% and 92% of the total components, respectively. Oxygenated monoterpene in coriander were the main metabolites, representing 98% of the total components. In carrot and celery, monoterpene and sesquiterpene hydrocarbons were the predominant metabolite classes. Oxygenated monoterpenes and monoterpene hydrocarbons were the major classes of metabolites detected in dill EO, where they represented 67.59 and 31.25% of the total compounds, respectively. Unlike the other *Apiaceae* species, cumin EO had three dominant classes of metabolites: monoterpene hydrocarbons (34.30%), aromatic hydrocarbons (32.92%), and hydrocarbons (20.19%).

**Figure 1:**
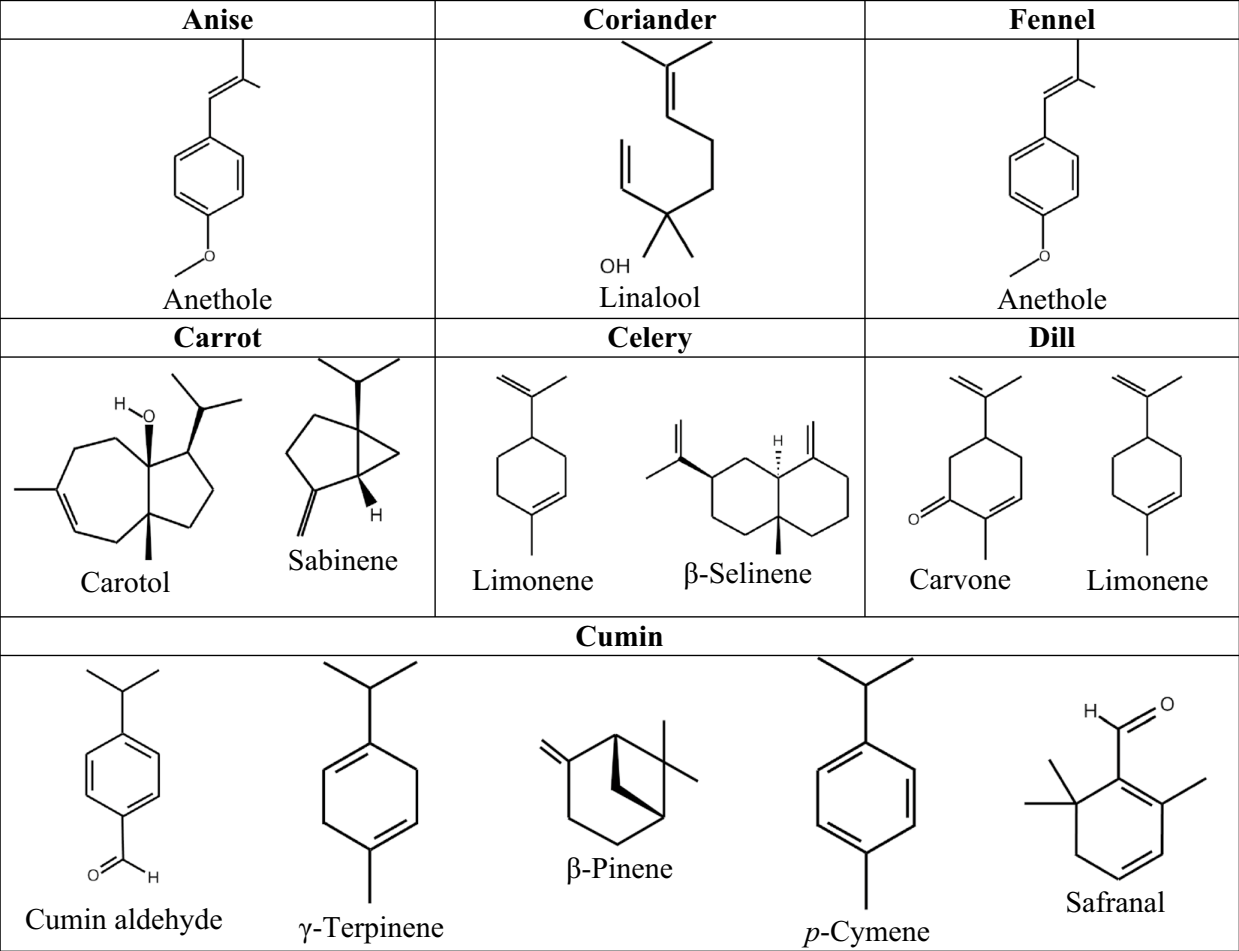
Structures of the most abundant components in anise, coriander, fennel, carrot, celery, dill and cumin.

These EOs showed a high degree of variability in volatile compounds composition (most abundant components chemical structures were given in Fig. 1). Total of 11 compounds were identified in anise EO, and the main component was anethole (93.74%). Thirteen compounds were identified in carrot EO, carotol (42.08%), sabinene (26.08%), β-pinene (7.97%), α-pinene (7.38%), and geranyl acetate (5.73%) represented the majority of the components. In celery EO, limonene (73.27%) and β-selinene (17.53%) were the major components, and total of 9 compounds have been identified in seed EO of celery. Carvone (67.41%) and limonene (30.58%) were the main compounds in dill out of 10 identified compounds in its EO. Coriander EO had two compounds, linalool (98.96%) and 3-hexyl hydroperoxide (1.04%). Similar to the anise EO, the main component in fennel EO was anethole (88.79%) followed by 4-anisaldehyde (2.95%) and methyl chavicol (2.83%). Fifteen compounds were identified in cumin EO, and cumin aldehyde (32.13%), γ-terpinene (20.19%), β-pinene (15.86%), *p*-cymene (14.47%), and safranal (9.76%) represented the predominant components. Fennel and cumin had the highest number of EO components identified among the examined species.

**Figure 2:**
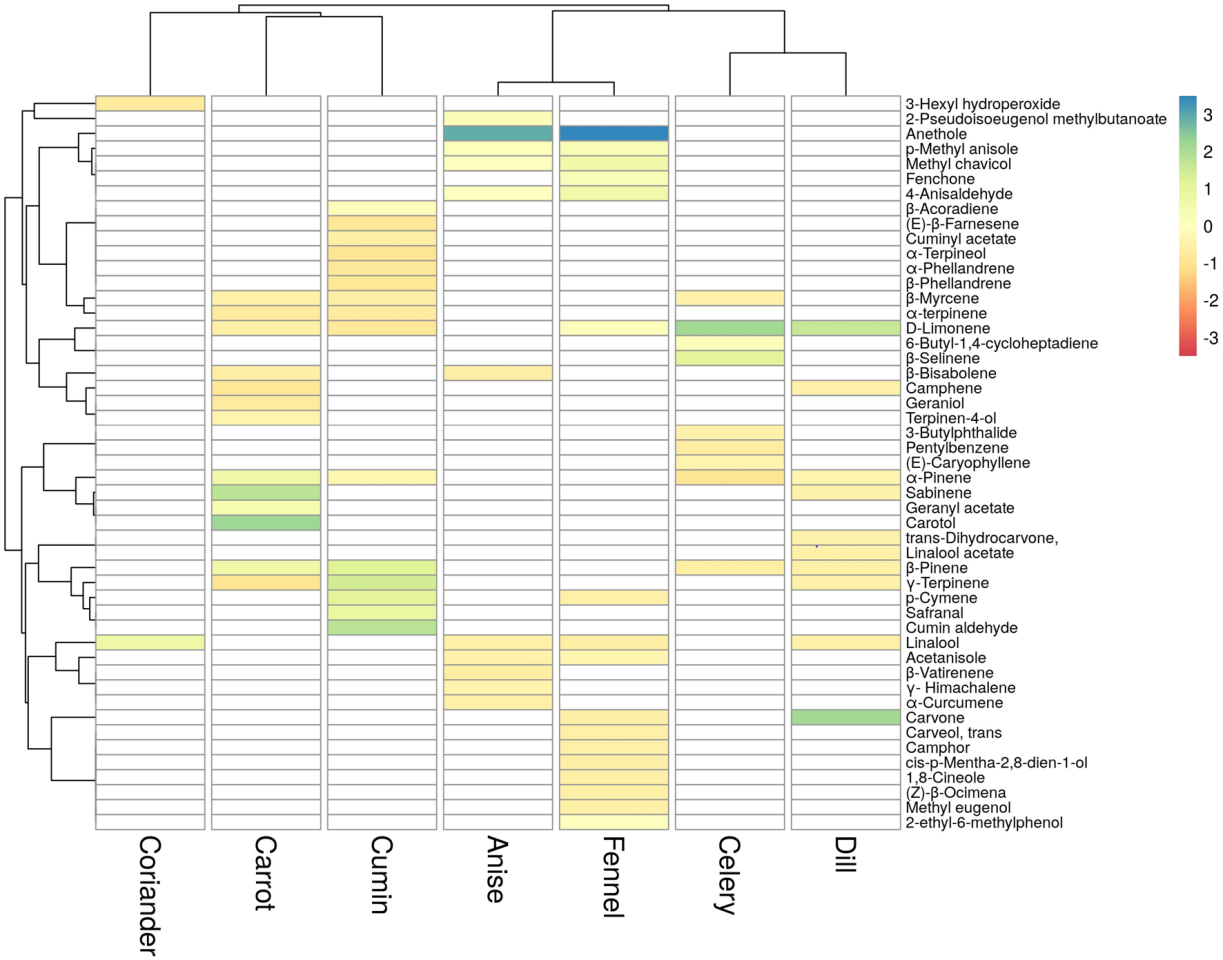
Cluster analysis generated by heatmap of EOs of different Apiaceae species based on the identification of compounds shown in Table 1. Blue boxes indicate higher, red boxes indicate lower concentrations than the mean.

Multivariate analysis was performed based on GC–MS results to determine the relationship and similarities between the species used in the study. The percentage of 49 EO compounds detected in the species was shown in Fig. 2 as a heatmap and as a dendrogram. The relative peak areas of all components in the samples were used to order species according to their affinity by subjecting them to HCA. Cluster analysis placed these species into four clusters: coriander as the first, carrot and cumin as the second, anise and fennel as the third and celery and dill as the fourth. Anise and fennel clustered within a group due to chemotaxonomic similarity of common compounds found in these species, such as anethole, *p-*methyl anisole, methyl chavicol and 4-anisaldehyde. Celery and dill were also clustered together due to the high concentration of limonene. Coriander was clustered separately because only two volatile compounds were detected and their quantity were different. The heatmap also showed that some of the EO components were species specific.

### Antioxidant activity of EOs

The in vitro antioxidant capacity of the EOs from the seeds was determined with two techniques (CUPRAC and DPPH), both of which are extremely sensitive methods with consistent results. The copper (II) ion-reducing ability of the EOs changed significantly among the species (Table 2). The CUPRAC activity in cumin, dill and celery EOs were 89.07, 14.66 and 13.83 µmol Trolox g^−1^, respectively, while the activity in coriander, fennel, carrot and anise EOs were 6.63, 6.49, 5.16 and 4.63 µmol Trolox g^−1^, respectively. The DPPH radical scavenging activity was lower than the CUPRAC activity. DPPH activity (µmol Trolox g^−1^) was 9.86 for dill, 4.18 for celery, 2.74 for cumin, 1.91 for anise, 1.73 for fennel, 1.18 for coriander and 0.26 for carrot, respectively.

**Table 2:** Antioxidant activities (CUPRAC and DPPH) of essential oils.

EO	CUPRAC (µmol Trolox g^−1^ EO)	DPPH (µmol Trolox g^−1^ EO)
Anise	4.63 ± 0.06 c	1.91 ± 0.02 d
Carrot	5.16 ± 0.18 c	0.26 ± 0.04 f
Celery	13.83 ± 0.52 b	4.18 ± 0.07 b
Dill	14.66 ± 0.40 b	9.86 ± 0.01 a
Coriander	6.63 ± 0.11 c	1.18 ± 0.06 e
Fennel	6.49 ± 0.24 c	1.73 ± 0.15 d
Cumin	89.07 ± 2.00 a	2.74 ± 0.08 c

### Antibacterial activity

**Figure 3:**
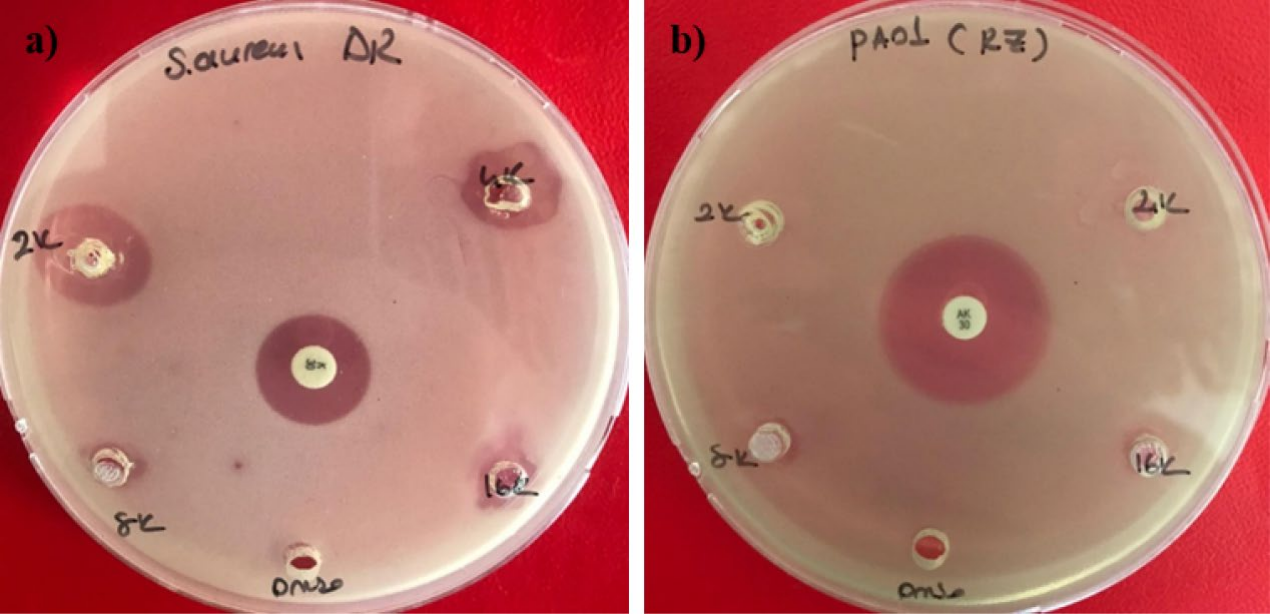
Agar well diffusion assay for dill (**a**) and fennel (**b**) EO against *S. aureus* and *P. aeruginosa* 2 k (50% v/v), 4 k (25% v/v), 8 k (12.5% v/v), 16 k (6.25% v/v), K30 (kanamycin), AK30(amikacin) and DMSO (5% v/v).

The in vitro antibacterial activity of the EOs were tested against the *S. aureus*, *B. cereus*, *E. coli,* and *P. aeruginosa.* Table 3 shows the inhibition zones of the EOs for the agar well diffusion assay. Coriander, cumin and dill EOs exhibited inhibitory effects against all tested strains except *P. aeruginosa,* while fennel and celery EOs were effective against *E. coli* and *B. cereus* strains, respectively (Fig. 3). Anise and carrot EOs did not show any antibacterial effect against the tested bacteria species.

**Table 3:** Inhibition zone diameters and MBC values of the essential oils (v/v %) against *S. aureus*, *B. cereus*, *E. coli*, and *P. aeruginosa* using the agar well diffusion method. *: > 4% (v/v) or no inhibition.

EO	Concentration (v/v)%	*S. aureus*	*B. cereus*	*E.coli*	*P. aeruginosa*
Coriander	50	> 30	17.3 ± 3.0	> 30	0
	25	> 30	18.3 ± 1.5	> 30	0
	12.5	11.8 ± 1.5	18.0 ± 1.2	18.6 ± 1.1	0
	6.25	8.3 ± 0.5	9.3 ± 0.5	14.0 ± 1.0	0
	MBC (v/v)%	0.06	0.25	0.25	*
Cumin	50	15.0 ± 1.0	11.6 ± 1.5	11.6 ± 0.5	0
	25	12.6 ± 2.3	10.6 ± 0.5	9.6 ± 0.5	0
	12.5	11.0 ± 1.0	9.3 ± 0.5	8.0 ± 0.0	0
	6.25	8.0 ± 0.0	8.3 ± 0.5	0	0
	MBC (v/v)%	0.5	0.25	1	*
Dill	50	14.0 ± 0.8	22.3 ± 2.5	10.6 ± 0.5	0
	25	12.3 ± 0.9	19.3 ± 1.1	9.6 ± 0.5	0
	12.5	0	0	8.0 ± 0.0	0
	6.25	0	0	0	0
	MBC (v/v)%	0.5	0.5	0.5	*
Fennel	50	0	0	9.3 ± 0.4	0
	25	0	0	8.0 ± 0.0	0
	12.5	0	0	0	0
	6.25	0	0	0	0
	MBC (v/v)%	0	*	4	*
Celery	50	0	14.6 ± 0.5	0	0
	25	0	9.6 ± 0.5	0	0
	12.5	0	8.3 ± 0.5	0	0
	6.25	0	0	0	0
	MBC (v/v)%	*	2	*	*
Anise	50	0	0	0	0
	25	0	0	0	0
	12.5	0	0	0	0
	6.25	0	0	0	0
	MBC (v/v)%	*	*	*	*
Carrot	50	0	0	0	0
	25	0	0	0	0
	12.5	0	0	0	0
	6.25	0	0	0	0
	MBC (v/v)%	*	*	*	*
	K30/AN30	14.6 ± 0.5	22.6 ± 0.5	15.0 ± 1.0	24.6 ± 0.4
	DMSO	*	*	*	*

Coriander EO (25%) exhibited the strongest antibacterial activity against *S. aureus*, *E. coli* (> 30 mm) and *B. cereus* (18.3 ± 1.5 mm). The MBC of the coriander EO for *S.* *aureus,* and *E. coli* were 0.06% (v/v) and 0.25% (v/v), respectively. The negative control, 5% DMSO, showed no inhibitory effect (Table 3). It should also be noted that coriander (25 and 50% (v/v)) EO showed a higher antibacterial effect than the kanamycin.

## Discussion

The chemical compositions of the EOs revealed both qualitative and quantitative variability. The EO yield after hydrodistillation of anise seeds was between 2 and 3%^4^, and anethole was the main component (79.0–92.9%), while methyl chavicol, anisaldehyde and bisabolene were also detected^25–27^. Similar EO profiles were identified in the current study, and anethole was the major compound (93.7%) of anise EO. Although EOs obtained from carrot flowers were investigated in several studies, the EO profile of carrot seeds was not studied adequately. While Özcan and Chalchat^28^ reported that the main components of carrot seeds were carotol, daucene and α-farnesene, the EO of the carrot seeds did not share any main compound other than carotol in the current study. Also, EOs of carrot seeds obtained from natural populations in Tunisia consisted mainly of monoterpenes (more than 50% of total oil) followed by sesquiterpenes (33.95–38.82%)^29^. Carrot seed EO in our study showed similar class compositions with a marked difference in the ratio of components. The major components of the EO obtained from celery seeds were limonene (73.27%) and β-selinene (17.53%). Presence of limonene and β-selinene in celery EO was reported by Khalid et al.^30^, Hassanen et al.^21^, and Zorga et al.^31^. Hydrodistillation of dill seeds yielded 2–4% oil, and the major compounds in the EO was carvone (47.7–73.6%) and limonene (12.4–16.6%)^4,32^ and major compounds in dill leaves were α-phellandrene, *p*-cymene and limonene^20^. In this study, carvone (67.41%) and limonene (30.58%) were also found to be the major compounds in dill seed EO. Hydrodistillation of coriander revealed the presence of 27 compounds, and the major compound was linalool (66.29%)^33^, and the presence of linalool as the major compound was confirmed by other reports^34,35^. Similarly, linalool (98.96%) was found to be the main compound present in coriander EO in this study. The EO yield of fennel seeds was between 2 and 4%^4^, and anethole was the major compound in fennel EO from different countries^36–39^, our results also showed that anethole was the main compound of fennel. However, estragole was also reported to be the main compound in fennel seeds from Tunus and Egypt^13,14,19^. Cumin EO contained cumin aldehyde, γ-terpinene, β-pinene and *p*-cymene as the major compenents^40^, but our results revealed presence of safranal and β-acoradiene as the other important compounds in cumin seed EO. Diverse volatile profiles of EOs could be influenced by plant part, development stage, harvest time, and the variability in climate and soil conditions. In addition, other abiotic and biotic factors could also alter expression of genes in pathways, resulting in the formation of diverse chemical patterns^41^.

*Apiaceae* is a taxonomically complex genus due to large number of species it contains. Hence the comparison of phytochemical profiles of anise, carrot, celery, dill, coriander, fennel and cumin is necessary. Multivariate analysis methods were effective in distinguishing related species^6,42^. The GC–MS results were subjected to HCA analysis and the samples were divided into four clusters according to their affinity to each other. These results show that chemotaxonomy studies could be carried out with EOs in *Apiaceae*.

Ethanolic, methanolic, acetone, oil and aqueous extracts of *Apiaceae* members were investigated for their antioxidant activities using free radical and superoxide anion radical scavenging activities^1^. It is recommended that at least two different methods should be employed for a reliable estimation of antioxidant capacity^43^. Therefore, antioxidant capacity of the EOs was evaluated by CUPRAC and DPPH methods. The antioxidant activity measured by the CUPRAC method was found to be higher than the results of the DPPH method. Phenolic compounds are among the secondary metabolites that can potently suppress free radicals. However, hydrocarbons also have roles in suppressing free radicals^6,44^. The highest hydrocarbon content was in cumin EO, and the high CUPRAC activity observed in the study may be related to high hydrocarbon content of cumin EO. The high antioxidant activity of cumin EO could also be related to the presence of other antioxidant compounds, such as cumin aldehyde and γ-terpinene^1^. Oxygenated compounds could also contribute overall antioxidant effects of EOs^6^, since Ferreira et al.^45^ reported that EOs with high oxygenated monoterpene compounds had high antioxidant capacity. Dill EO had a moderate hydrocarbon and oxygenated monoterpene content and showed the highest DPPH activity. The difference between the CUPRAC and DPPH results could raise from their transfer mechanisms^46^, DPPH reaction uses hydrogen atom transfer mechanism^47^, and CUPRAC is mainly based on electron transfer mechanism. In addition, DPPH uses only a radical dissolved in organic solvent, therefore valid for hydrophobic systems^48^. Therefore, CUPRAC is more sensitive technique to evaluate antioxidant activity.

Coriander, cumin and dill EOs showed antibacterial activity against three bacteria, but fennel and celery EOs showed antibacterial activity only against *E. coli* and *B. cereus*, respectively. Although, fennel seed EO was only effective against *E. coli,* fennel leaf and seed EOs were shown to be effective different bacteria and fungus spepces^17,19^. Coriander oil at 25% concentration exerted promising activity comparable to other tested EOs with > 30 mm inhibition zone diameters against Gram-positive *S. aureus* and Gram-negative *E. coli* strains. The reported antibacterial activity of the coriander, cumin and dill EOs is consistent with previously published data^20,49–53^. Although many studies have been conducted to investigate the antibacterial activity mechanisms of EOs, it is difficult to attribute the activity to a single component^6^. There are many studies reported that the antibacterial properties of these EOs are related to the activity of the major components, and the resulting synergistic or antagonistic effects. In the present study, the potent antibacterial activity of coriander EO is apparently due to its major compound linalool (98.96%), which with known antibacterial properties^54–56^. The antibacterial activity of cumin EO is attributed to cuminaldehyde (32.13%), γ-terpinene (20.19%), β-pinene (15.86%), and *p*-cymene (14.47%), which inhibit the bacterial growth^57,58^. The antibacterial activity of dill EO is perhaps related to the its carvone (67.41%), and limonene (30.58%) content, which have been reported to possess antibacterial properties^59,60^. Carrot and anise EOs, which had carotol and anethol as their major compounds, did not show any antibacterial activity against the tested bacteria.

## Conclusions

The chemical profiles, antioxidant capacity and antibacterial activity of seven EOs obtained from anise, carrot, celery, dill, coriander, fennel and cumin were investigated. Total of 49 compounds were identified in seed EOs. Monoterpene hydrocarbons were the predominant chemical compounds in carrot, celery and cumin, oxygenated monoterpenes in dill and coriander, and phenylpropanoids in anise and fennel EOs. HCA analysis showed close relationships between celery with dill, anise with fennel, and cumin with carrot. The highest CUPRAC and DPPH activity was found in cumin and dill EOs, respectively, which also had moderate antibacterial activity. Coriander EO showed moderate CUPRAC and low DPPH activity, while exhibiting high antibacterial activity. Due to high antibacterial activities of coriander, cumin and dill EOs against the bacteria, they could potentially be used as natural antibacterial agents. However, effects of individual compounds, mechanisms of actions, safety and toxicity should be studied further to have a better understanding to be able to use in food industry.

## Materials and methods

### Plant material

The seeds of anise, coriander, fennel and cumin were obtained growing and harvesting local genotypes in research farms of Isparta University of Applied Sciences (37° 45′ N and 30° 33′ E, 997 m). Carrot, celery and dill seeds were obtained from a commercial grower from Ardıçlı village (37° 48′ N and 30° 12′ E, 906 m) of Isparta.

### Isolation and analysis of essential oils

Anise, carrot, celery, dill, coriander, fennel and cumin EOs were isolated for 3 h using a Neo-Clevenger type hydrodistillation. Triplicate seed samples were weighted and EOs from each replicate were extracted for each species separately, and the EOs obtained from each replicate were used for further analysis. The EO contents were measured as a percentage (v/w). The isolated samples were dried out over anhydrous sodium sulphate and were stored in a sealed vial at 4 °C before GC–MS analysis. GC–MS and GC-FID analyses of EOs were performed using a Shimadzu 2010 Plus with QP-5050 quadrupole detector equipped with a Rxi®-5Sil MS (30 m × 0.25 mm, 0.25 μm) capillary column and CP-Wax 52 CB (50 m × 0.32 mm; film thickness 0.25 μm), respectively. The injector temperature was set at 250 °C, the initial column temperature was held at 60 °C for 3 min, then gradually increased to 220 °C at 10 °C min^−1^, and finally held for 10 min at 220 °C. The injection volume was 1 μL. The carrier gas was helium with a flow rate of 2.0 mL min^−1^ and a split ratio of 1:20. The percent composition of the identified components was calculated from the GC peak areas without correction factor. The mass spectra were compared with the mass spectra of the Wiley, Flavors and Fragrances of Natural and Synthetic Compounds (FFNSC) and the National Institute of Standards and Technology libraries (NIST Tutor). The quantitative determination was conducted using Gas Chromatography/Flame Ionization Detector (GC-FID), Shimadzu Model operating at the same conditions of GC–MS. The EOs (50 μL) was solubilized in 5 mL of n-hexane and injected into the split mode 1/100. The main components of each EO (anethole, caratol, sabinene, α-pinene, limonene, linalool, cumin aldehyde, γ-terpinene, β-pinene and p-cymene) were injected into the GC device as standard for identification and quantification. The identification of the components was finally confirmed by comparing their retention indices with those of the authentic compounds.

### Antioxidant activities of the essential oils

Each of the EO (500 μL) was weighed and dissolved in 9.5 mL of absolute ethanol. Antioxidant activities were determined from the diluted samples. The cupric reducing antioxidant capacity (CUPRAC) and 2,2-diphenyl-1-picryl-hydrazyl-hydrate (DPPH) radical-scavenging assays were performed to determine the in vitro antioxidant activity of the EOs. The CUPRAC activity of EOs was performed according to method of Apak et al.^61^. CUPRAC reactions were set up as follows: 1 mL of 0.01 M copper (II) chloride, 1 mL of 0.0075 M neocuproine solution and 1.0 mL of 1 M ammonium acetate buffer solution (pH 7.0) were added successively into a glass tube. All solutions were prepared in absolute ethanol. Subsequently, the appropriate amount of extract solution was added and total reaction volume was brought to 4.1 mL with absolute ethanol and mixed well. Absorbance against a reagent solution without a sample was measured at 450 nm after 30 min. Tests analysis were performed in triplicate and CUPRAC activity was calculated as Trolox equivalents per g of EO (µmol Trolox g^−1^ EO), through a calibration curve with Trolox standard.

$$CUPRAC \left( {\mu mol TR g^{ - 1} } \right) = \frac{A}{{\varepsilon_{TR} }} \times \frac{{V_{m} }}{{V_{s} }} \times D_{f} \times \frac{{V_{E} }}{m} \times 1000$$

where *A*: Sample absorbance measured at 450 nm; *Ɛ*_*TR*_: molar absorption coefficient of Trolox compound in the CUPRAC method (1.67 × 10^4^ L mol^−1^ cm^−1^); *Vm*: Total volume of CUPRAC method measuring solution; *Vs*: Sample volume (mL); *Df*: Dilution factor; *V*_*E*_: Volume of the prepared extract (mL); *m*: The amount of EO taken in the extraction process (g).

DPPH radical-scavenging activity of the EOs was measured according to Bener et al.^62^ Reactions for DPPH method were set up as follows: X mL of extract solution, “2 − X” mL 99% ethanol and 2 mL of 0.2 mM of DPPH· solution were added to a glass tube and mixed well. The reaction mixture was maintained at room temperature for 30 min in the dark and the absorbance was measured at 515 nm against ethanol with a spectrophotometer (Shimadzu UV-1280, Kyoto, Japan). Reactions were performed in triplicate and DPPH activity was expressed as Trolox equivalents per g of EOs (µmol Trolox g^−1^ EO) and calculated according to the following equation:

$$DPPH \left( {\mu mol TR g^{ - 1} } \right) = \frac{{\Delta_{A} }}{{\varepsilon_{TR} }} \times \frac{{V_{m} }}{{V_{s} }} \times D_{f} \times \frac{{V_{E} }}{m} \times 1000$$

where *Ɛ*_*TR*_: molar absorption coefficient of Trolox compound in the DPPH method (2.16 × 10^4^ L mol^−1^ cm^−1^); *V*_*m*_: Total volume of DPPH method measuring solution; *V*_*s*_: Sample volume (mL); *Df*: dilution factor; *V*_*E*_: Volume of the prepared extract (mL); *m*: The amount of EO taken in the extraction process (g).

### Antibacterial activities of essential oils

#### Bacterial strains

Gram-positive strains *Bacillus cereus* (ATCC 11,778), *Staphylococcus aureus* (ATCC 25923) and Gram-negative strains *Pseudomonas aeruginosa* (PA01)*, Escherichia coli* (ATCC 25922) were used to test antimicrobial properties of the EOs.

#### Agar well diffusion

The antibacterial activities of the EOs were evaluated using an agar well diffusion assay^63^. A 100 μL bacterial culture (corresponding to 1.5 × 10^8^ CFU mL^−1^) of *S. aureus*, *B. cereus*, *E. coli,* and *P. aeruginosa* were added in soft Luria Bertani Agar (LBA) (containing 0.5% agar) and poured into sterile petri dishes. The wells (6 mm diameter) were made with a sterile cork borer after solidification of LB agar plates. Then, dilutions of each EO ranging from 6.25 to 50% (v/v) was made with dimethyl sulfoxide (DMSO) and added to the wells (50 μL). The plates were incubated at 37 °C for 24 h. Antibacterial activity was evaluated by measuring the diameters (mm) of the clear zone of growth inhibition. Kanamycin (K 30 µg) (for *E. coli, B. cereus* and *S. aureus)* and Amikacin (AN 30 µg) (for *P. aeruginosa*) were used as positive controls, and DMSO was used as a negative control. All assays were conducted in triplicate in three independent experiments.

#### Determination of the minimum bactericidal concentration

The minimum bactericidal concentration (MBC) of the tested EOs were evaluated using the broth dilution method. Each bacterial culture was prepared and adjusted to 0.5 McFarland (containing about 1.5 × 10^8^ CFU mL^−1^). Each EO was diluted with DMSO, and added to each tube to achieve the final test concentrations from 4.0 to 0.0625% (v/v).

The bacterial suspensions were added to obtain a final population of about 10^6^ CFU mL^−1^. An aqueous solution of 5% DMSO was used as a negative control. Tubes were incubated for 24 h at 37 °C. To determine the MBC values, an aliquot of 5 µL from each tube that did not show an apparent turbidity was cultured on LB agar plates and incubated at 37 °C for 24 h. The MBC was determined as the lowest concentration where no growth was visually observed. All assays were conducted in triplicate in three independent experiments.

### Statistical analysis

The experiments were carried out in a completely randomized design, with three replications for each analysis. Data were subjected to analysis of variance (ANOVA) using SPSS Statistics 22.0 software (IBM, Armonk, NY, USA). Tukey test (*P* ≤ 0.05) was used to separate differences between the means. The results were given as mean ± standard deviation. Hierarchical Cluster Analysis (HCA) and the corresponding heatmap were prepared using the ClustVis online tool^64^.

### Ethical approval

The collection of plant material and the performance of experimental research on such plants complied with the national guidelines of (Türkiye).

## Data Availability

The datasets generated during and/or analyzed during the current study are available from the corresponding author on reasonable request.
