# Publisher Correction: Chemical composition and biological activities of essential oils of seven Cultivated *Apiaceae* species

**DOI:** 10.1038/s41598-024-64032-5

**Published:** 2024-06-25

**Authors:** Sercan Önder, Çağdaş Deniz Periz, Seyhan Ulusoy, Sabri Erbaş, Damla Önder, Muhammet Tonguç

**Affiliations:** 1 Department of Agricultural Biotechnology, Faculty of Agriculture, Isparta University of Applied Sciences, 32200 Isparta, Türkiye; 2 Department of Biology, Faculty of Engineering and Natural Sciences, Süleyman Demirel University, 32260 Isparta, Türkiye; 3 Department of Field Crops, Faculty of Agriculture, Isparta University of Applied Sciences, 32200 Isparta, Türkiye

Correction to: *Scientific Reports* 10.1038/s41598-024-60810-3, published online 02 May 2024

The original version of this Article contained errors in Table 1, where the values under the "Class compositions" heading were incorrectly positioned. The original Table 1 and accompanying legend appear below.

**Table 1:** Chemical components of essential oil in seeds of the seven Apiaceae isolated by hydrodistillation.

No	RI^a^	RI^lit^	Components	Formula	Class	Volatile oils (%)						
						Anise	Carrot	Celery	Dill	Coriander	Fennel	Cumin
1	926	930*	3-Hexyl hydroperoxide	C_6_H_14_O_2_	H	–	–	–	–	1.04 ± 0.03	–	–
2	936.1	939*	α-Pinene	C_10_H_16_	MH	–	7.38 ± 0.12	0.13 ± 0.001	0.37 ± 0.001	–	–	1.62 ± 0.14
3	950.3	954*	Camphene	C_10_H_16_	MH	–	0.57 ± 0.001	–	0.12 ± 0.001	–	–	–
4	973.0	975*	Sabinene	C_10_H_16_	MH	–	26.08 ± 1.15	–	0.14 ± 0.001	–	–	–
5	977.7	978**	β-Pinene	C_10_H_16_	MH	–	7.97 ± 0.41	0.82 ± 0.001	0.04 ± 0.001	–	–	15.86 ± 0.53
6	989.2	990*	β-Myrcene	C_10_H_16_	MH	–	1.25 ± 0.07	1.21 ± 0.01	–	–	–	0.76 ± 0.04
7	1004.1	1002*	α-Phellandrene	C_10_H_16_	MH	–	–	–	–	–	–	0.35 ± 0.01
8	1017.1	1059*	α-terpinene	C_10_H_16_	MH	–	0.91 ± 0.02	–	–	–	–	0.60 ± 0.03
9	1023.8	1018**	*p*-Methyl anisole	C_8_H_10_O	ArHy	1.51 ± 0.03	–	–	–	–	1.74 ± 0.15	–
10	1024.3	1028*	*p*-Cymene	C_10_H_14_	MH	–	–	–	–	–	0.03 ± 0.001	14.47 ± 0.42
11	1029.5	1029*	Limonene	C_10_H_16_	MH	–	1.26 ± 0.08	73.27 ± 2.31	30.58 ± 1.02	–	0.66 ± 0.07	0.39 ± 0.01
12	1030.0	1020***	β-Phellandrene	C_10_H_16_	MH	–	–	–	–	–	–	0.25 ± 0.001
13	1031.8	1034*	1,8-Cineole	C_10_H_18_O	OM	–	–	–	–	–	0.10 ± 0.001	–
14	1037.8	1039*	(Z)-β-Ocimene	C_10_H_16_	H	–	–	–	–	–	0.04 ± 0.001	–
15	1059.7	1052**	γ-Terpinene	C_10_H_16_	H	–	1.50 ± 0.09	–	0.17 ± 0.001	–	–	20.19 ± 0.69
16	1087.6	1078*	Fenchone	C_10_H_16_O	OM	–	–	–	–	–	1.46 ± 0.11	–
17	1099	1085**	Linalool	C_10_H_18_O	OM	0.06 ± 0.002	–	–	0.04 ± 0.001	98.96 ± 2.43	0.02 ± 0.001	–
18	1131.4	1103*	cis-*p*-Mentha-2,8-dien-1-ol	C_10_H_16_O	OM	–	–	–	–	–	0.04 ± 0.001	–
19	1143.4	1146*	Camphor	C_10_H_16_O	OM	–	–	–	–	–	0.05 ± 0.001	–
20	1143	1074***	Pentylbenzene	C_11_H_16_	ArHy	–	–	0.59 ± 0.07	–	–	–	–
21	1146	1149****	6-Butyl-1,4-cycloheptadiene	C_11_H_18_	H	–	–	4.03 ± 0.13	–	–	–	–
22	1177.1	1164**	Terpinen-4-ol	C_10_H_18_O	OM	–	1.69 ± 0.14	–	–	–	–	–
23	1189.7	1174***	α-Terpineol	C_10_H_18_O	OM	–	–	–	–	–	–	0.18 ± 0.001
24	1195.8	1200*	Methyl chavicol	C_10_H_12_O	P	1.13 ± 0.09	–	–	–	–	2.83 ± 0.24	–
25	1200	1270****	Safranal	C_10_H_14_O	OM	–	–	–	–	–	–	9.76 ± 0.26
26	1201.4	1192*	*trans*-Dihydrocarvone	C_10_H_16_O	OM	–	–	–	0.10 ± 0.001	–	–	–
27	1217.1	1122****	*trans*-Carveol	C_10_H_16_O	OM	–	–	–	–	–	0.05 ± 0.001	–
28	1237.9	1283****	Cumin aldehyde	C_10_H_12_O	ArHy	–	–	–	–	–	–	32.13 ± 1.21
29	1242.0	1205***	Carvone	C_10_H_14_O	OM	–	–	–	67.41 ± 1.24	–	0.03 ± 0.001	–
30	1251.8	1248****	4-Anisaldehyde	C_8_H_8_O_2_	AlA	1.18 ± 0.12	–	–	–	–	2.95 ± 0.31	–
31	1254.9	1242****	Geraniol	C_10_H_18_O	OM	–	1.45 ± 0.07	–	–	–	–	–
32	1255.2	1248****	Linalyl acetate	C_12_H_20_O_2_	OM	–	–	–	0.04 ± 0.001	–	–	–
33	1285.2	1255*	Anethole	C_10_H_12_O_2_	P	93.74 ± 1.15	–	–	–	–	88.79 ± 1.71	–
34	1348	1345****	Acetanisole	C_9_H_10_O_2_	ArAl	0.20 ± 0.001	–	–	–	–	0.31 ± 0.001	–
35	1379.9	1351****	Geranyl acetate	C_12_H_20_O_2_	OM	–	5.73 ± 0.24	–	–	–	–	–
36	1401.8	1369****	Methyl eugenol	C_11_H_14_O_2_	P	–	–	–	–	–	0.09 ± 0.001	–
37	1420.1	1412***	(E)-Caryophyllene	C_15_H_24_	SH	–	–	1.27 ± 0.02	–	–	–	–
38	1432	1428*	Cuminyl acetate	C_12_H_16_O_2_	ArHy	–	–	–	–	–	–	0.79 ± 0.003
39	1455.9	1450***	(E)-β-Farnesene	C_15_H_24_	SH	–	–	–	–	–	–	0.24 ± 0.001
40	1465.5	1459****	β-Acoradiene	C_15_H_24_	SH	–	–	–	–	–	–	2.31 ± 0.16
41	1475.9	1482*	γ- Himachalene	C_15_H_24_	SH	0.28 ± 0.001	–	–	–	–	–	–
42	1482.2	1481****	α-Curcumene	C_15_H_22_	SH	0.06 ± 0.001	–	–	–	–	–	–
43	1485	1478*	2-ethyl-6-methylphenol	C_9_H_12_O	P	–	–	–	–	–	0.81 ± 0.009	–
44	1486	1479**	β-Vatirenene	C_15_H_22_	SH	0.05 ± 0.001	–	–	–	–	–	–
45	1486.1	1517***	β-Selinene	C_15_H_24_	SH	–	–	17.53 ± 0.91	–	–	–	–
46	1508.4	1504***	β-Bisabolene	C_15_H_24_	SH	0.01 ± 0.001	1.17 ± 0.08	–	–	–	–	–
47	1595.3	1595****	Carotol	C_15_H_26_O	SH	–	42.08 ± 1.12	–	–	–	–	–
48	1654	1649**	3-Butylphthalide	C_12_H_14_O_2_	ArHy	–	–	1.06 ± 0.03	–	–	–	–
49	2567	1848*	2-Pseudoisoeugenol	C_15_H_20_O_3_	ArHy	1.75 ± 0.001	–	–	–	–	–	–
Class compositions												
Aliphatic aldehyde (AlA)	1.18 ± 0.04	–	–	–	–	2.95 ± 0.24	-					
Monoterpene hydrocarbon (MH)	–	45.42 ± 1.34	75.43 ± 2.74	31.25 ± 0.79	–	0.69 ± 0.03	34.30 ± 1.12					
Aromatic alcohol (ArAl)	0.20 ± 0.007	–	–	–	–	0.31 ± 0.01	–					
Aromatic hydrocarbon (ArHy)	3.26 ± 0.17	–	1.65 ± 0.08	–	–	1.74 ± 0.11	32.92 ± 0.89					
Oxygenated monoterpene (OM)	0.06 ± 0.001	8.87 ± 0.12	–	67.59 ± 2.22	98.96 ± 1.12	1.75 ± 0.21	9.94 ± 0.13					
Sesquiterpene hydrocarbon (SH)	0.40 ± 0.009	43.25 ± 1.23	18.80 ± 0.42	–	-	–	2.55 ± 0.09					
Hydrocarbon (H)	–	1.50 ± 0.07	4.03 ± 0.13	0.17 ± 0.008	1.04 ± 0.02	0.04 ± 0.001	20.19 ± 0.75					
Phenylpropanoid (P)	94.87 ± 2.74	–	–	–	–	92.52 ± 2.51	–					
Total	99.97	99.04	99.91	99.01	100	100	99.90					
Number of components	11	13	9	10	2	17	15					

^a^Retention index were determined on a Restek Rxi®-5Sil MS column using a set of standards consisting of C_6_-C_30_ *n*-alkanes; -:Not detected. *^19^,**^22^,***^23^,****^24^.

The original article has been corrected.

